# Reliability and Validity Assessment of Mizaj Questionnaire: A Novel Self-report Scale in Iranian Traditional Medicine

**DOI:** 10.5812/ircmj.15924

**Published:** 2014-03-05

**Authors:** Morteza Mojahedi, Mohsen Naseri, Reza Majdzadeh, Mansoor Keshavarz, Mohammad Ebadini, Esmaeil Nazem, Mohsen Saberi Isfeedvajani

**Affiliations:** 1 Department of Iranian Traditional Medicine, Faculty of Medicine, Shahed University, Tehran, IR Iran; 2 Traditional Medicine Clinical Research Center, Shahed University, Tehran, IR Iran; 3 Department of Epidemiology and Biostatistics, School of Public Health and Knowledge Utilization Research Center, Tehran University of Medical Sciences, Tehran, IR Iran; 4 Department of Physiology, School of Medicine, Tehran University of Medical Sciences, Tehran, IR Iran; 5 Department of Iranian Traditional Medicine, Tehran University of Medical Sciences, Tehran, IR Iran; 6 Medicine, Quran and Hadith Research Center and Department of Community Medicine, Faculty of Medicine, Baqiyatallah University of Medical Sciences, Tehran, IR Iran

**Keywords:** Medicine, Traditional, Unani, Temperament, Questionnaires, Reproducibility of Results

## Abstract

**Background:**

In Iranian Traditional Medicine, mizaj (temperament) plays a key role in preventive, therapeutic and lifestyle recommendations. A reliable self-reported scale for mizaj identification is critically needed to introduce ITM into the official medical and health care system especially in the case of designing national preventive protocols.

**Objectives:**

The present study aimed to design a preliminary self-administered mizaj questionnaire and assessed its reliability and validity in Iran.

**Patients and Methods:**

In this cross-sectional study, a questionnaire with 52 items was designed based on mizaj-related indices. Subsequent to content and face validity assessment, using qualitative and quantitative method, 47 items remained. Based on the non-randomly sampling, the test-retest reliability of each question and internal consistency of the questionnaire was examined by the participation of 35 volunteers. The reliable version questionnaire was filled up by 52 volunteers wherein they were divided into warm/cold and wet/dry groups based on their mizaj which was predetermined by a team of expert practitioners. Logistic regression analysis was performed for validity process between the experts’ assessment of mizaj and each of the items in the questionnaire that resulted to the final ten-item questionnaire divided into two subscales. By using ANOVA and post Hoc with Dunnet statistics, the optimum cut-off points were defined and their sensitivity and specificity was assessed.

**Results:**

The weighted kappa coefficients of the 39 items were between 0.40 and 0.82 showing their acceptable reliability and the Cronbach’s α coefficient was 0.71 showing the internal consistency. The sensitivity and specificity of the final questionnaire cut-off points were 65% and 93% for the warm group, 52% and 97% cold group, 53% and 67% dry group and finally 53% and 76% wet group.

**Conclusions:**

Our results suggested that many of the designed questions according to the literature’s mizaj identification indices had satisfactory reliability and the final ten-item questionnaire could discriminate the different groups of mizaj, therefore, this can be used as the first version of a brief self-report mizaj estimating scale.

## 1. Background

In modern medicine, it is well known that healthy individuals have different physical and mental characteristics and there is a genetic variation within and between the races (1). This can also be deduced from the point that different paraclinical findings and anthropometric indices have a wide normal spectrum in healthy individuals (2-6). Most of the traditional medical schools such as the traditional Chinese medicine (TCM), Ayurveda and specially the Iranian traditional medicine (ITM) have based their preventive orders and diagnosis and treatment decisions on the discrimination of these differences (7-12). Nowadays, conventional medicine is also heading towards personalizing medicine and paying attention to individual differences in the pathogenesis, progression of diseases and response to therapeutics (13-16). Metabonomics, nutrigenomics and also pharmacogenetics that try to classify individuals according to their possible response to medicine are the new promising areas of personalized medicine (5, 16, 17). The school of ITM which originated from the ancient Iranian civilization was established upon the basic concept of mizaj (temperament) (18, 19). Basically mizaj is developed due to the interaction of different elements in the human body and affects the normal physical and emotional characteristics and also the physiological functions of the body (20, 21). According to this concept, each person has a unique characteristic named mizaj which is recognized and classified by his or her morphological, physiological and psychological features (20, 22). According to ITM, a person is considered to be in a healthy state when his or her mizaj keeps its balance and most of the diseases occur when the mizaj becomes imbalanced (21). It is believed that the number of mizaj equals to the number of living individuals in the world (10, 12). Since the numerous number of mizaj may be imagined, the elites of ITM have divided all kinds of mizaj into nine major groups for easy assessment. These nine groups may be imagined as the sectors of two dimensional spectrums of different degrees of warmness and wetness. These sectors includes one central equilibrium or medium region and eight circumferential, out of the equilibrium regions which consist of four simple mizajes (warm, cold, moist, and dry) and four combined mizajes (warm and moist, warm and dry, cold and moist, cold and dry). In the context of this theory, each member of these groups is susceptible to certain diseases related to his or her mizaj and may need different treatment for the same disease and even different lifestyle recommendation for health care and disease prevention (21-23). In other words, the mizaj acts as a road map for the maintenance of individuals’ health. For example, an individual with a cold and moist mizaj is recommended to have more physical activity than the warm and dry etc. (22, 24). Individuals are sorted into these nine major groups of mizaj by the use of mizaj identification criteria (23). Eminent ITM scholars defined these criteria and described the indices of each criterion in their literatures but most of these indices are qualitative and their discrimination capabilities have not been assessed (10, 22, 23, 25).

To the best of our knowledge of the published articles, there is no standard method or tool available for the determination of the mizaj to be used in research and clinical practice (26-30). On the other hand, no relevant study was found to investigate the relationship between each claimed mizaj index and the variety of mizaj to be considered as healthy status. For the above mentioned reason, today’s ITM practitioners also estimate the mizaj of the individuals by their own personal impression, thus, sometimes the mizaj of a person is reported differently by two different practitioners (31, 32). An objective and reliable measurement scale that has been developed through a scientific method based on the concept of mizaj is absolutely necessary to fulfill the scientific research requirements in ITM. Besides, designing such a questionnaire to become self-reportable and user-friendly can help healthy individuals determine their mizaj on their own and subsequently accustom their health care and lifestyle orders according to ITM (31, 33). To meet these scientific requirements and public demands, we aimed to take a step to objectify and quantify the subjective and qualitative mizaj indices by designing a preliminary questionnaire for mizaj determination in healthy young individuals and subsequently to assess its reliability and validity.

## 2. Objectives

This study was the first research that carried out the preliminary steps of developing an objective and easily applied scale for mizaj determination in ITM. We designed a preliminary self-administered questionnaire according to mizaj indices and assessed its reliability and validity.

## 3. Patients and Methods

The procedure of this cross-sectional study included four steps: item generation, recruitment of participants, validity process and scoring.

### 3.1. Item Generation

Credible ITM textbooks were assessed by our research team and their opinions on the mizaj indices were compared. At last, Avicenna’s book on "The Canon of Medicine" was selected as the original reference. In the next process, we studied the relative chapters of the book word by word and extracted all items that were relevant to the mizaj identification (22). The indices described by the items for inclusion had to be of persistent characteristics and appropriate for a self-report questionnaire. The initial questions were designed based on the subjective parameters using the help of our initial expert panel comprising of three expert practitioners who had more than 10 years of experience in ITM. The items were Likert-type having five options representing five diverse points on a bilateral spectrum relating to each index. We decided to arrange the options in an order that relate the first options to complete coldness or wetness, the second to partial coldness or wetness, the third to equilibrium mizaj, the fourth to partial warmness or dryness and finally the fifth referred to complete warmness or dryness (22, 34, 35). Considering their application for different groups of general population and investigators, the mizaj questions and their answers were translated into colloquial language so that it could be easily understood.

### 3.2. Recruitment of Participants

Our study samples were selected non-randomly. To study the reliability and validity of the questionnaire respectively, 50 and then 150 students of Tehran University of Medical Sciences (located in Tehran, capital of Iran) were invited to participate (36-38). We only included the subjects meeting the following criteria of being normal healthy individuals according to the modern medicine and ITM, in the range of 20-40 years of age from either sex that voluntarily agreed to participate in the study by giving their informed consent. The participants were assured that their information will remain confidential. The volunteers with the history of chronic diseases, sustained drug use, cigarette smoking or being pregnant or in the lactation or menstruation period at the time of the study were excluded. The above screening process was performed by three ITM practitioners; one of them also had professional knowledge and training in modern medicine.

### 3.3. Validity Process

In order to determine content and face validity, our second expert panel members including 10 other ITM practitioners, 3 ITM expert practitioners and 7 ITM practitioners with professional knowledge and training of modern medicine who also had more than 10 years of clinical experience were invited and informed about the study aims and procedures. In order to equalize the experts’ conceptions of content validity indices (relevancy, clarity and comprehensiveness of the questionnaire), the definition of these indices were explained to them (39-42). The ability of designed questions to reflect the content was defined as relevancy. The questions lucidity concerning their wording and concept was considered as clarity. Finally, the questionnaire’s ability to include all content domains was defined as comprehensiveness (40, 43). Upon the panel’s agreement, the initial questions were mailed to them and each expert was asked to write his or her additional comments about the items. Moreover, we asked them to share their opinions on the extracted indices and to suggest some questions which they believed were appropriate for mizaj identification. After collecting the experts’ opinions, the initial expert panel modified some of the questions based on the feedbacks (39, 40). In the next step, the questions were assessed by the cooperation of 40 volunteers from different groups of people; they completed the questionnaire and wrote their opinions in relation to any difficulty felt in the comprehension of the questions or answers. At these stages, the face validity of the questionnaire was also evaluated (44). Finally, the items in need of revision were re-worded in order to be grammatically and colloquially acceptable and easily comprehended. We sent back the corrected questionnaire to the secondary panelists for another round asking them to indicate their degree of agreement on the relevancy and clarity of each item and also the comprehensiveness of the questionnaire (40, 44). They were asked to rate each item’s clarity and reliability and also the comprehensiveness of the questionnaire from 1 to 4 (1 = inappropriate, 2 = somewhat appropriate, 3 = appropriate, 4 = quite appropriate). The experts’ answers were collected and the content validity indices were calculated. To determine the inter-rater agreement (IRA) for the relevancy and clarity of each item, the number of experts who chose "quite appropriate" or "appropriate" for each item was divided by the total number of experts. In this stage, the items were retained if their item content validity index (I-CVI) were more than or equal to 0.7, indicating acceptable agreement (42, 43). The next version of the questionnaire was formed after the deletion of nonrelated questions based on their low content validity index (I-CVI < 0.7). The IRA for the relevancy and clarity of the new questionnaire was estimated with the scale content validity index (S-CVI). To estimate the S-CVI, we calculated S-CVI/Ave averaging the I-CVIs by summarizing them and dividing by the number of items. The questionnaire’s comprehensiveness was achieved via dividing the number of experts who chose the comprehensiveness of the questionnaire "quite appropriate" or "appropriate" by the total number of experts (40, 43). This questionnaire is in Persian and can be completed in 10-20 minutes. For the reliability process, the first group of volunteers (50 participants) was invited and the preliminary questionnaire was filled up by the qualified participants twice, at an interval of 2-3 weeks. The recruiting and survey implementation period took time from February to May 2012 and the location of study was Tehran University of Medical Sciences in Iran. The test-retest was performed to verify the reliability of each question (42, 45). Subsequently the proportion of observed agreement and the weighted kappa coefficient of each question were calculated. Finally, the questions which had acceptable weighted kappa coefficients (≥ 0.40) were selected for designing a reliable questionnaire and the Cronbach’s α coefficient of the recent questionnaire was calculated (46-48). For validity assessment, the second group of volunteers (150 participants) was invited to enroll in the study. Each eligible participant completed the reliable questionnaire and subsequently was visited by 4-8 expert ITM physicians or practitioners to separately determine his or her mizaj. This step was conducted during June and July 2012 in Tehran University of Medical Sciences in Iran. We gathered the results of the expert’s diagnosis, assessed their agreement and then selected the mizaj status of the participants with acceptable agreement (≥ 70%) in their mizaj identification, as our Gold Standard (37, 47). For the validity analysis, we had to dichotomize the answers of each item and also the results of mizaj diagnosis made by the experts into warm/non-warm, cold/non-cold, wet/non-wet and dry/non-dry groups. Following that we applied two methods, firstly estimating the sensitivity, specificity and eventually Youden index (J) of each item (45, 49), secondly, we used separate binary logistic regression models for each group using forward stepwise (50, 51). We defined the questions as acceptable valid items if they had J ≥ 0.2 and were proven significantly (P < 0.2) in at least one model of binary logistic regression (37, 45, 50). We also conformed if the results of the sensitivity and specificity evaluation were based more on three main options of questions as compared to five option, we deleted two partial options in final questionnaire for easy application.

### 3.4. Scoring

A brief questionnaire based on the above mentioned valid items was extracted and its items were divided into two subscales (warm/cold and wet/dry). We calculated the total scores of the warm and cold group in the warm/cold subscale as well as the wet and dry group in the wet/dry subscale and used one-sample Kolmogorov-smirnov Test (KS). If the variables distribution was normal according to KS, we used ANOVA and post Hoc with Dunnet statistic; otherwise, we used Kruskal-Wellis test for the comparison of total score means between groups (51, 52). We examined several cut-off point values for each subscale and calculated their sensitivity, specificity and likelihood ratios. Finally, we selected the best cut-off point according to the total score of 95% confidence interval in each warm, cold, wet and dry group (52). The statistical analysis was performed using the STATA version 10 and the SPSS version 16.

## 4. Results

### 4.1. Items Generation

According to "The Canon of Medicine", Avicenna has divided individuals of general population to nine major groups based on their mizaj and described ten criteria (Ajnas-e-Ashara) to sort them into these nine groups. These criteria included the characteristics of a person in relation to touch, muscle and fat mass, hair condition, skin color, physique, speed of impressibilities, sleep and wakefulness, physical functions, quality of waste matter and last but not the least, the psychic functions. He has also defined different subjective and objective indices for each criterion. We extracted and summarized the details of these ten criteria and their corresponding indices in Table 1 as our first result.

**Table 1.** The Mizaj Identification Criteria and Indices According to "The Canon of Medicine"

Criterion	Indices			
****	warmness	coldness	wetness	dryness
**Touch**	warm skin	cold skin	soft nails	hard nails
	-	-	soft skin	fragile nails
	-	-	smooth skin	hard skin
	-	-	-	coarse skin
**Muscle and fat mass**	muscular	fatty	muscular or fatty	thin
**Hair condition**	rapid growth	slow growth	slow growth	rapid growth
	bushy	scanty	straight	curly
	thick	thin	grey or white	-
	curly	straight	-	-
	black	reddish, brown, grey or white	-	-
**Skin color**	red, yellow or brunette	white, gloomy, purple, chalky or ivory	white, chalky or ivory	purple or gloomy
**Physique**	vast chest	small chest	wide nose	prominent joints
	large extremities	small extremities	non prominent joint	prominent adam's apple (larynx cartilage)
	wide vessels	narrow vessels	-	thin and straight nose
	prominent vessels	hidden vessels	-	-
	large pulse	small pulse	-	-
	strong pulse	weak pulse	-	-
**Impressibility speed**	rapidly impressed from warmness and warm natured foods	rapidly impressed from coldness and cold natured foods	-	-
**Sleep and wakefulness**	more wakeful	more sleepy	more sleepy	more wakeful
**Physical functions**	rapid growth	slow growth	-	-
	strong voice	weak voice	-	-
	loud voice	low voice	-	-
	rapid speech	slow speech	-	-
	continuous speech	articulate speech	-	-
	swift movement	slow movement	-	-
	rapid blinking	slow blinking	-	-
**Quality of waste matter (stool, urine. sweat)**	strong odor	weak odor	-	-
	dark color (yellow or red)	light color	-	-
**Psychic function**	strong rage	weak rage	rapid disappearance of reactions (anger, pleasure, etc.)	persistence of reactions
	rapid rage	slow rage	unstable imaginations	stable imaginations
	rapid comprehension		weak memory	strong memory
	brave	slow comprehension	-	-
	optimistic	timid	-	-
	hopeful	pessimistic	-	-
	happy	hopeless	-	-
	lively	sad	-	-
	high moral	weary	-	-
	not much impressible	low moral	-	-
	cruel	very impressible	-	-
	dream of fire & sun	kind	-	-
		dream of water& ice	-	-

### 4.2. Content and Face Validity Results

The extracted indices resulted in the design of a preliminary questionnaire with 52 questions which subsequently was reduced to 47 questions after content validity analysis. The CVI of each recent question was between 0.70 and 1.00, indicating that most of the ITM experts agreed with the selected items and their related questions. The inter-rater agreement (IRA) for the relevancy, clarity, and comprehensiveness of the final 47 items questionnaire were 82.76%, 78.72%, and 80%, respectively.

### 4.3. Items Reliability Analysis

Out of the 50 invited participants, 4 were excluded as they were using medications, 6 were eliminated by our practitioners due to their illnesses or imbalanced mizaj and finally 5 refused to cooperate. In conclusion, 35 participants were able to accomplish both assessments. The mean age was 28.2 ± 7.3 years, 14 (40%) males and 21 (60%) females. The average time for completing the questionnaire for each person was 15 minutes (range: 10 to 20 minutes) and the participants had no difficulty in answering the questions and from their points of view, there were no unclear questions in the questionnaire. The final result of this stage was a 39-item questionnaire with satisfactory reliability reached after the estimation of the observed agreement and weighted kappa coefficient in the test-retest. The weighted kappa coefficient of 20 questions were between 0.40-0.59, 18 questions were between 0.6-0.79 and one question was 0.83 (See Appendix 1 and 2). The Cronbach’s α coefficient of this questionnaire was 0.71. These 39 reliable questions were selected for validity assessment.

### 4.4. Items Validity Analysis

Among the second group of participants (150 volunteers), 14 volunteers had illnesses or other exclusion criteria and 15 could not complete the study, therefore, 121 participants were visited by expert practitioners; whereas, 52 persons had acceptable agreement (≥ 70%) in their mizaj detection between 4-8 practitioners. The mean age was 20.9 ± 1.2 years, 26 (50%) males and 26 (50%) females. Based on the results of analysis, in the warm group, Q9, Q10, Q11, Q12, Q13, Q16, Q19, Q20, Q22, Q25, Q26, Q30 and Q33 had J ≥ 0.2 and Q1, Q11, Q16 and Q25 were significant in the final warm model of logistic regression. In the cold group, Q1, Q16, Q17, Q20, Q22, Q24, Q25 and Q31 had J ≥ 0.2 and also Q1, Q17, Q24 and Q25 were significant in the final cold model of logistic regression. In the wet group, only Q3 had J ≥ 0.2 that was also significant in the final wet model according to binary logistic regression. Finally, Q2, Q5, Q8, Q9 and Q10 had J ≥ 0.2 in the dry group and Q2, Q3 were significant items in the final dry model of logistic regression. Further details are presented in Table 2. 

**Table 2.** Selected Questions With High Youden Index (J ≥ 0.2) or Being Significant in Different Logistic Regression Models ^a^

Item	Mizaj Group Diagnosed by Expert Practitioners											
	Warm Group			Cold Group			Wet Group			Dry Group		
	J	Warm Model ^b^		J	Cold Model ^c^		J	Wet Model ^d^		J	Dry Model ^e^	
		OR	P Value		OR	P Value		OR	P Value		OR	P Value
**Q1**	0.19	6.9	0.029	0.36	24.1	0.002	-	-	-	-	-	-
**Q2**	-	-	-	-	-	-	-	-	-	0.5	4.6	0.050
**Q3**	-	-	-	-	-	-	0.17	8.5	0.065	-	12	0.001
**Q5**	-	-	-	-	-	-	-	-	-	0.23	-	-
**Q8**	-	-	-	-	-	-	-	-	-	0.21	-	-
**Q9**	0.33	-	-	-	-	-	-	-	-	0.21	-	-
**Q10**	0.2	-	-	-	-	-	-	-	-	0,25	-	-
**Q11**	0.32	13.5	0.026	-	-	-	-	-	-	-	-	-
**Q12**	0.28	-	-	-	-	-	-	-	-	-	-	-
**Q13**	0.2	-	-	-	-	-	-	-	-	-	-	-
**Q16**	0.32	7.4	0.041	0.22	-	-	-	-	-	-	-	-
**Q17**	-	-	-	0.28	3.7	0.185	-	-	-	-	-	-
**Q19**	0.21	-	-	-	-	-	-	-	-	-	-	-
**Q20**	0.3	-	-	-	-	-	-	-	-	-	-	-
**Q22**	0.33	-	-	0.3	-	-	-	-	-	-	-	-
**Q24**	-	-	-	0.3	24	0.014	-	-	-	-	-	-
**Q25**	0.34	4.06	0.083	0.29	9	0.021	-	-	-	-	-	-
**Q26**	0.31	7.6	0.026	-	-		-	-	-	-	-	-
**Q30**	0.8	-	-	-	-		-	-	-	-	-	-
**Q31**	-	-	-	0.82	-		-	-	-	-	-	-
**Q33**	0.24	-	-	-	-		-	-	-	-	-	-
**Q39**	-	-	-	-	-		0.26		-	-	-	-

^a^ Abbreviations: OD, odds ratio.

^b^ P < 0.001 , correction=76.9%.

^c^ P < 0.001, correction = 90.4%.

^d^ P = 0.036, correction = 69%.

^e^ P < 0.001, correction = 75%.

### 4.5. Scoring and Validation of 10 Items Brief Questionnaire

Based on prior results, we proposed a brief 10-item questionnaire and assessed its validity. Among them, 8 items (Q1, Q11, Q16, Q17, Q20, Q24, Q25 and Q26) were related to warmness/coldness named warm/cold subscale and 2 items (Q2 and Q3) belonged to wetness/dryness named wet/dry subscale. According to KS test, in the warm/cold subscale, the distribution of total score was normal (P < 0.05) and based on Post Hoc test with Dunnett statistics, there were significant differences between the warm and cold group (P < 0.001).

In the wet/dry subscale, the KS test did not provide normal distribution, but based on Kruskal-Wellis test, there were significant differences between the wet (CI 95% = 10.3-17.1) and dry (CI 95% = 21.2-29.6). According to this scale, each person had two scores, one for warm/cold subscale that ranged from 8 to 24 and one for wet/dry subscale that ranged from 2 to 6. The best cut-off point values (warm ≥ 19 & cold ≤ 14 for warm/cold subscale and dry ≥ 5 & wet ≤ 3 for wet/dry subscale) were selected. The sensitivity and specificity of the questionnaire based on selected cut-off points were 65% and 93% for warm group, 52% and 97% cold group, 53% and 67% dry group and finally, 53% and 76% wet group [ Appendix 3 ]. The mean and standard deviation (SD) of each item has been brought in Appendix 4.

## 5. Discussion

Since diagnosis, treatment, prevention and health care orders of ITM depends on each person’s individual mizaj, ITM has an individualized model and can be viewed as a "personalized medicine" (14, 21, 31). Since the range of mizaj identification indices described in the eminent ITM textbooks are much outspread and most of them are qualitative, inconsistencies exist among ITM practitioners and also general population to determine the mizaj (19, 32). As other alternative medicine also encounter this problem and their diagnostic concepts are not sufficiently verified (53-55), researchers are trying to develop standard tools for diagnostic protocols (56-58). Altogether, quantifying and objectifying the qualitative and subjective indices and diagnostic symptoms are important concerns of integrative eastern medicine (21, 27, 56, 59). Although recent studies in ITM have attempted to objectify and quantify mizaj identification indices but it seems that there is no reliable measurement tool available in the published articles, consequently, our study can be considered as the first study in this concept (26-28, 60). The first aim of our study was to design a prototype mizaj identification questionnaire through a scientific method and the second aim was to assess the reliability and the validity of the designed items as a preliminary step of developing a mizaj determination standard tool (39, 48). For the first stage, we selected "The Canon of Medicine" as the base reference, because of its comprehensibility and good classification of indices. Other ITM textbooks had similar sentences about mizaj identification indices and there was no considerable difference between them (10, 23, 25, 61). Most of the experts who participated in the study had professional knowledge and training in both ITM and modern medicine and this was an advantage in our study but considering that our study was an exploratory study to design a reliable objective self-report questionnaire, our team made great efforts to describe the aim and method to the ITM experts in order to familiarize them with the epidemiological methodology.

Based on the results of reliability analysis, one question (Q3) had weighted kappa coefficient of above 0.80 (0.83). This question was about obesity and based upon "The Canon of Medicine" and the experts’ viewpoint, it was an important index to assess wetness or dryness (22). On the other hand, it was selected as an appropriate diagnostic item for wetness and dryness in the final questionnaire. Since some anthropometric indices like this had maximum reliability and validity in our study and are approximately stable over the years - despite other short-interval varying indices-we recommend further studies to assess the exact validity of these indices. The weighted kappa coefficients of 18 questions were substantial (0.60-0.79). These questions included two questions about touch characteristics, two were related to muscle and fat mass, four mentioned hair condition, one was about physique, five belonged to physical functions, three were related to psychic function and one question was about the quality of waste matter. Hence, eight out of the ten criteria (Ajnas-e-Ashara) had substantial reliable questions as representatives in our questionnaire, although the two remaining criteria meaning the speed of impressibilities and sleep/wakefulness had moderate weighted kappa coefficient (0.40-0.59) [ Appendix 1 ]. Since in "The Canon of Medicine", each criterion had unequal number of indices in comparison to the other criteria; our designed questions were unequal in number ranging from one question for skin color to 9 questions for psychic function. Among the eight unreliable remaining items (kappa < 0.4) some answers such as dreams are more influenced by extraneous factors and naturally we had expected their low reliability. But about the answers such as nail condition which is constant over the months, furthermore studies with different questions may need to evaluate their repeatability and also their correlation with mizaj status. In the validity process, we considered the questions as valid items if they not only had J ≥ 0.2 but also were present in the related logistic regression model. By the above consideration, we were able to arrange each eligible item into the appropriate group. Hence, six items (Q1, Q11, Q16, Q20, Q25, Q26) were set in the warm group, four items (Q1, Q17, Q24, Q25) were placed in the cold group, the Q2 and Q3 were put in the dry group and finally Q3 was related to the wet group. Although two of the selected items had a J lower than 0.2 (Q1 = 0.19 in warm model and Q3 = 0.17 in wet model), they were considered eligible because of their short distance from the acceptable J. We refused to assess the equilibrium or medium group of each quality (warm/cold and wet/dry) because the number of participants whom had been diagnosed by experts to have equilibrium mizaj was very small to be suitable for analysis. Since it seems that the reliability and validity of mizaj identification indices were not assessed before; we did not have any similar data to compare our results with. In 2008, Shahabi et al. assessed the healthy persons of hot and cold nature (mizaj) in terms of changes in their neuroendocrine and immune systems. They claimed that they used a standard questionnaire for temperaments and nature determination of the subjects, but they did not mention their reference or their scientific method (27). They also divided the healthy individuals to four mizaj groups of Choleric, Sanguine, Phlegmatic and Melancholic. This kind of division has inconsistency with Avicenna and the other ITM Elites’ opinion of nine group division explained previously in the introduction and mostly referred it to the dominance of the humors in pathologic conditions (imbalanced mizaj) (19, 22). Furthermore, some of the used items in the mentioned questionnaire such as digestive symptoms, rash, epistaxis etc. are pathologic signs and symptoms that occur in the disease and imbalanced mizaj (22, 23, 25). In 2011, Dar et al. claimed that they divided their selected volunteers into different groups according to their temperaments, but they did not mention their measures or selection method (26). Other available studies had similar conditions (9, 60). We also did not suggest weighted scores for final scale because the sensitivity and specificity of the non-weighted and weighted scores were close to each other yet further studies need to assess the preference of either score. Although this study suggested a reliable and valid brief user-friendly self-report scale for mizaj identification, there were some limitations or weaknesses that could be considered for future studies. The main limitation of the study was that there was no suitable "Gold Standard" for validation assessment. Other studies in Eastern Traditional Medicine have had the same limitations (53, 55, 56). To treat this defect, we enrolled 4-8 expert practitioners to diagnose the participant’s mizaj but there was no acceptable agreement between them and we had to acquiesce to 70% or upper agreement between the practitioners. In future studies, serious efforts must be made to solve this problem. The small number of diagnosed participants with high practitioner agreement about their mizaj was another limitation and weakness of our study. Because the small number of participants (52 persons), in comparison to the large number of questions (39 questions), we had to refuse some relevant analysis such as explanatory factor analysis (EFA) and confirmatory factor analysis (CFA). In addition, there were a small number of diagnosed participants in the equilibrium group then we had to exclude this important group. This matter decreased the validation of the study because to the best of our knowledge this group must have gathered the most number of participants in itself (22). We suggest more studies to make an attempt to solve this defect especially by encouraging practitioners to spend more time to this kind of study and through focus group discussions with the participation of expert practitioners to increase their diagnostic agreement (35, 61). Another limitation of our study was the elimination of some details of mizaj indices in order to avoid questionnaire elongation, moreover, since the purpose of this study was to assess mizaj indices through a brief self-administered questionnaire, we only selected the subjective parameters to design the questionnaire and therefore, the objective indices such as pulse diagnosis were omitted. Single direct questions for some of the indices, capable of influencing the answers were also a limitation; therefore, it is recommended to design more various indirect questions to assess the above-mentioned indices (35, 62). Because our sampling method was non-randomly, generalizability of our samples to the general population was less than random sampling. We suggest random sampling for further studies (37). Finally, the strong point of this study was the opening of a new way to objectify diagnostic indices in ITM and also the last questionnaire we named "Mizaj questionnaire, Version 1" is the first self-report scale with remarkable sensitivity and specificity in ITM, therefore, our study is the first step to developing standard scales in ITM.

As a conclusion, this study was the first research that carried out the preliminary steps to developing an objective and easily applied scale for mizaj determination in ITM. Our results showed that most of the questions designed based on the mizaj identification indices on "The Canon of Medicine" had reasonable reliability and that the selected valid items we suggested for the final questionnaire could validate further development of a standard self-reported mizaj determination scale. Further studies should be made to develop creditable versions of self-report mizaj identification scale to measure the healthy status based on ITM in general population to apply healthy protocols, basic research and student education.

**Appendices**

## Appendices

**Appendix 1.** The Reliable Questionnaire With Each Question's Observed Agreement and Weighted Kappa Coefficient ^a^

Question		Observed Agreement	Weighted Kappa	Lower CI*	Upper CI
**Q1**	When others touch your skin, What do they say about its warmness or coldness?	90	0.64	0.43	0.86
**Q2**	How is the condition of your skin's softness and dryness?	92.14	0.71	0.47	0.95
**Q3**	Are you fat or thin compared to others?	95.71	0.83	0.61	1.03
**Q4**	How is the portion of your muscles compared to fat tissues in your body?	94.29	0.76	0.55	0.97
**Q5**	How is the measure of your hair growth compared to the others in your age?	93.57	0.71	0.50	0.92
**Q6**	How much is the amount of your hair compared to others?	87.86	0.56	0.34	0.77
**Q7**	How is the thickness of your hair compared to others?	92.86	0.63	0.40	0.85
**Q8**	How is your hair condition compared to others?	94.29	0.77	0.56	0.99
**Q9**	Your hair color is in what range?	90.71	0.61	0.39	0.83
**Q10**	Your skin color is in what range?	87.14	0.65	0.38	0.91
**Q11**	How big is the palm of your hand?	90.71	0.55	0.33	0.77
**Q12**	How big is your foot compared to the others?	89.29	0.53	0.31	0.75
**Q13**	How is the width of your feet compared to the others?	92.86	0.66	0.44	0.89
**Q14**	How is the condition of Adam's apple compared to the soft tissue around it?	84.29	0.46	0.25	0.66
**Q15**	How is your nose shape?	87.14	0.41	0.20	0.63
**Q16**	How fast are you influenced by warmness and coldness?	80.71	0.40	0.20	0.61
**Q17**	How fast are you influenced by warm nature foods as honey, spices, paper or cold nature foods as buttermilk, yogurt and cucumber?	86.43	0.49	0.29	0.70
**Q18**	If you have no limit to sleep, how much sleep do you need in a day?	91.43	0.46	0.21	0.72
**Q19**	How was your growth rate in youth compared to the others?	90.71	0.60	0.37	0.82
**Q20**	How is your voice power compared to others?	90.71	0.59	0.36	0.82
**Q21**	How is the modulation of your voice?	92.14	0.64	0.40	0.88
**Q22**	How is the level of your voice tone?	87.14	0.48	0.26	0.70
**Q23**	How do you pronounce the words when you are talking?	89.29	0.60	0.37	0.84
**Q24**	How do you pronounce several consequent sentences?	84.29	0.46	0.23	0.68
**Q25**	How is your rage and anger?	86.43	0.60	0.38	0.81
**Q26**	How is your physical movements compared to others?	92.14	0.71	0.48	0.94
**Q27**	How is your blinking rate compared to others?	92.86	0.70	0.49	0.90
**Q28**	What color is your morning urine in a normal condition?	92.14	0.70	0.47	0.93
**Q29**	How is your urine smell in a normal condition?	89.29	0.53	0.30	0.76
**Q30**	What color is your feces in a normal condition?	88.57	0.42	0.18	0.66
**Q31**	How is your feces smell in a normal condition?	89.29	0.54	0.31	0.77
**Q32**	What color is your sweat in normal condition?	90	0.59	0.35	0.82
**Q33**	How much do you perceive your will power is compared to others in daily activities?	88.57	0.55	0.34	0.77
**Q34**	How is your reading comprehension compared to others?	89.29	0.58	0.36	0.79
**Q35**	How is your courage compared to others?	90	0.61	0.40	0.83
**Q36**	How is your pessimism and optimism compared to others?	90.71	0.55	0.32	0.78
**Q37**	How much is your hope to future?	87.14	0.45	0.24	0.66
**Q38**	How is your spiritual liveliness?	87.86	0.41	0.18	0.63
**Q39**	How is your lenity or cruelty compared to others?	93.57	0.707	0.48	0.93

^a^ Abbreviations: CI, confidence interval.

**Appendix 2.** Selected Items for Self-report Mizaj Questionnaire

Selected Items for Wetness or Dryness (Wet-dry Scale) ^a^				
		1	2	3
**Q1**	When others touch your skin, what do they say about its warmness or coldness?	cold	not cold, not warm	very warm
**Q2 ** ^**b**^	How is the condition of your skin's softness and dryness?	very soft	not soft, not dry	very dry
**Q3 ** ^**b**^	Are you fat or thin compared to others?	very fat	not fat, not thin	very thin
**Q11**	How big is the palm of your hand?	small	not small, not big	big
**Q16**	How fast are you influenced by warmness and coldness?	I feel cold, fast.	I feel the same in both cases.	I feel warm, fast.
**Q17**	How fast are you influenced by warm nature foods as honey, spices, paper or cold nature foods as buttermilk, yogurt and cucumber?	I feel cold, fast by cold nature foods	I feel the same in both cases.	I feel warm, fast by warm nature foods.
**Q20**	How is your voice power compared to others?	weak	not weak, not strong	strong
**Q24**	How do you pronounce several consequent sentences?	articulate	not articulate, not continuous	continuous
**Q25**	How is your rage and anger?	I get angry late	I get angry no late no fast	I get angry fast
**Q26**	How is your physical movements compared to others?	very slow	not slow, not fast	fast

^a^ The score of warm-cold scale could be 8 to 24. Warm ≥ 19, cold ≤ 14.

^b^ The score of wet-dry scale could be 2 to 6. Dry ≥ 5, wet ≤ 3.

**Appendix 3.** Analysis of Selected Cutoff Points of Warm-cold and Wet-dry Scales ^a^

Domain, Score	Sen	Spe	J	Prevalence	PPV	NPV	LR^+^	LR^-^
**Warm-cold scale**								
Warm ≥ 19	65	93	0.58	44	88	77	9.46	0.37
Cold ≤ 14	52	97	0.49	44	92	72	15.13	0.5
**Wet-dry scale**								
Dry ≥ 5	53	67	0.2	37	48	71	1.58	0.71
Wet ≤ 3	53	76	0.29	37	56	74	2.17	0.63

^a^ Abbreviations: LR, likelihood ratio; NPV, negative predictive value; PPV, positive predictive value; Sen, sensitivity; spe, specificity.

**Appendix 4.** The Mean and Standard Deviation (SD) of Each Item in the Final Questionnaire

Item	Total, Mean ± SD	Warm Group, Mean ± SD	Cold Group, Mean ± SD	Dry Group, Mean ± SD	Wet Group, Mean ± SD
**Q1**	2.102 ± 0.80	2.392 ± 0.66	1.742 ± 0.81	-	-
**Q11**	2.042 ± 0.62	2.302 ± 0.63	1.872 ± 0.55	-	-
**Q16**	1.902 ± 0.77	2.262 ± 0.75	1.602 ± 0.66	-	-
**Q17**	2.022 ± 0.70	2.172 ± 0.58	1.782 ± 0.73	-	-
**Q20**	2.332 ± 0.73	2.562 ± 0.66	2.132 ± 0.81	-	-
**Q24**	2.132 ± 0.69	2.392 ± 0.50	1.912 ± 0.79	-	-
**Q25**	2.192 ± 0.89	2.602 ± 0.66	1.912 ± 0.95	-	-
**Q26**	2.132 ± 0.79	2.432 ± 0.73	1.862 ± 0.76	-	-
**Q2**	1.852 ± 0.87	-	-	2 ± 1	1.682 ± 0.82
**Q3**	2.362 ± 0.66	-	-	2.792 ± 0.42	2 ± 0.67
